# The Dark Triad in Managers: Expression and Its Association with Leadership Style

**DOI:** 10.1192/j.eurpsy.2026.11899

**Published:** 2026-07-31

**Authors:** S. D. Tomilovskiy, A. M. Konovalova

**Affiliations:** 1 Faculty of Psychology, Lomonosov Moscow State University; 2 Department of Pedagogy and Medical Psychology, Sechenov First Moscow State Medical University, Moscow, Russian Federation

## Abstract

**Introduction:**

The Dark Triad refers to a cluster of three interrelated personality traits – Machiavellianism, narcissism, and psychopathy – which can significantly influence professional behavior. In managerial contexts, these traits often manifest in decision-making strategies, interpersonal attitudes, and preferred leadership styles.

**Objectives:**

To examine differences in the expression of Dark Triad traits among managers at various organizational levels and to analyze their association with leadership styles.

**Methods:**

The study involved 30 managers from different levels of organizational responsibility.

Dark Triad traits were assessed using the “Dirty Dozen” questionnaire.

Leadership styles were evaluated with the Management Decision-Making Styles Scale (Fetiskin et al.).

The relationship between variables was calculated using Pearson’s correlation coefficient.

**Results:**

Managers at the highest organizational level showed the highest expression of Dark Triad traits, particularly in combination with situational and implementer leadership styles.

In contrast, middle- and lower-level managers predominantly demonstrated a permissive leadership style, which was associated with lower Dark Triad scores.

Correlation analysis revealed:

- a positive association between the implementer style and narcissism;
- a negative relationship between the permissive style and all three Dark Triad traits;
- a moderate positive association between the situational style and Machiavellianism.

**Conclusions:**

The findings suggest that certain Dark Triad traits may function as adaptive mechanisms in managerial contexts and are linked to more flexible and outcome-oriented leadership styles, particularly at higher organizational levels.

**Disclosure of Interest:**

None Declared

